# Defect Detection and Segmentation Framework for Remote Field Eddy Current Sensor Data

**DOI:** 10.3390/s17102276

**Published:** 2017-10-06

**Authors:** Raphael Falque, Teresa Vidal-Calleja, Jaime Valls Miro

**Affiliations:** Centre for Autonomous Systems (CB 11.09.300), Faculty of Engineering and Information Technology, University of Technology Sydney, 15 Broadway, Ultimo, NSW 2007, Australia; teresa.vidalcalleja@uts.edu.au (T.V.-C.); jaime.vallsmiro@uts.edu.au (J.V.M.)

**Keywords:** Remote Field Eddy Current (RFEC), Non-Destructive Evaluation (NDE), defect segmentation, active-contour

## Abstract

Remote-Field Eddy-Current (RFEC) technology is often used as a Non-Destructive Evaluation (NDE) method to prevent water pipe failures. By analyzing the RFEC data, it is possible to quantify the corrosion present in pipes. Quantifying the corrosion involves detecting defects and extracting their depth and shape. For large sections of pipelines, this can be extremely time-consuming if performed manually. Automated approaches are therefore well motivated. In this article, we propose an automated framework to locate and segment defects in individual pipe segments, starting from raw RFEC measurements taken over large pipelines. The framework relies on a novel feature to robustly detect these defects and a segmentation algorithm applied to the deconvolved RFEC signal. The framework is evaluated using both simulated and real datasets, demonstrating its ability to efficiently segment the shape of corrosion defects.

## 1. Introduction

Water pipelines made of ferromagnetic materials are subject to corrosion, which can lead to expensive and dangerous pipe failures. These failures can be predicted by assessing the quality of the pipelines and thus the infrastructure can be replaced before it happens. To assess the quality of the pipelines, the water industry relies on Non-Destructive Evaluation (NDE) technologies which allows for a cost-effective inspection. The Remote Field Eddy Current (RFEC) is one of the electromagnetic NDE technologies dedicated to the assessment of ferromagnetic pipes. This technology is deployed by means of an inline tool that travels inside a pipeline while gathering information related to the remaining non-corroded pipe wall thickness. The analysis of the data gathered by the tool plays a critical role for the timely replacement of the corroded pipes sections, ultimately avoiding pipe failures.

**Figure 1:**
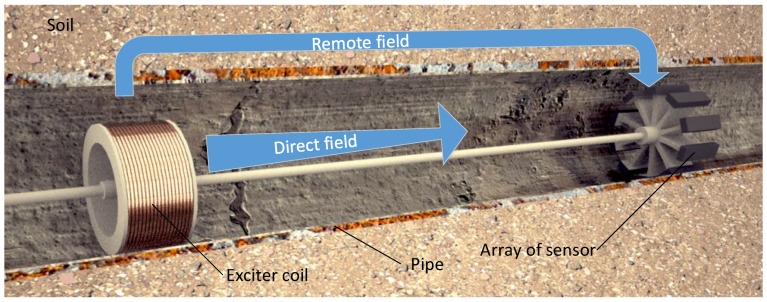
Schematic of the RFEC tool considered in this article. The exciter coil generates an electromagnetic field which is expelled outwards from the pipe near the exciter coil—hence the direct field is quickly attenuated. At a remote distance along the axial direction, the remote field flows inwards to the pipe and is measured by an array of receivers.

RFEC tools were originally designed with an exciter coil and a receiver coil located at a remote distance along the axial direction [1]. It can be easily shown through Finite Element Analysis (FEA) that the electromagnetic field generated by the exciter coil flows outwards from the pipe near the exciter coil and flows inwards to the pipe at a remote location, hence the placement of the receiver coil in this remote area [2]. This phenomenon, referred as *double through wall penetration* in the literature, is summarized in Figure 1. Each time the electromagnetic field crosses the pipe wall, its amplitude is attenuated and its phase is delayed proportionally to the non-corroded thickness of the pipe wall [3], making this technology particularly relevant for NDE. As a result, the sensor measurements of an RFEC tool are generally the amplitude of the electromagnetic field measured by the receiver coil and the phase-shift between the generated and measured electromagnetic field.

This article considers the task of automatically recovering the shape and extent of corrosion patches, cracks or pitting with an automatic and systematic approach. In this article the extraction of the extent and shape are referred as defect segmentation. To recover the extent and shape of a defect, it is important to have information on both the axial and radial directions. Thus, there is a need for a tool with multiple receivers distributed along the circumference—such as the one shown in Figure 1 with an array of receivers in the remote area. More information of such a tool regarding its practical implementation is discussed in [4]. The extraction of the depth of the defect has been studied in the literature [5] and is out of the scope of this work.

To the best of the authors’ knowledge, no work focused on extracting the extent and shape of defects using RFEC data has been published in the literature. The closest work consists of defect sizing (depth and extent) in the case of 2D axisymmetric geometries [5,6]. The common practice for extracting the shape of corrosion or other defects from data obtained with other NDE technologies, is to consider the task as an image segmentation problem. Segmentation methods have been considered for Magnetic Flux Leakage (MFL), and Pulsed Eddy Current (PEC) data—both electromagnetic NDE technologies used for pipeline assessment—where a comparison between region growing, minimum error thresholding, and morphological segmentation has been done on simulated data in [7]. As shown in the paper, region growing outperforms the other methodologies; unfortunately, this approach cannot be directly applied on raw RFEC data due to the *double through wall phenomenon* and the presence of Bell and Spigot (B&S) joints.

In fact, the *double through wall phenomenon* induces a signal convolution, where the changes in the sensor measurements are due to the change in thickness at, mainly, two different areas of the pipe—near the exciter coil, and near the receiver coil. This signal convolution—also referred to as signal shadowing—is discussed in the literature for tools with different receiver configurations; for axisymmetrical tools with a single receiver coil [8], for multiple receiver coils distributed along the axial direction [9] and for tools with an array of receivers [10]. Removing this shadowing effect and accounting for the B&S joints allows the use of classical image segmentation algorithms such as the one evaluated in [7].

In this work, we present a fully automatic framework for defect shape extraction from pipes inspected with RFEC sensors. To account for shadowing effects and the joints, the framework is subdivided in multiple tasks; (1) sensor measurement deconvolution based on the proposed optimization algorithm in [10], (2) B&S joint detection to locate independent pipe segments, (3) defect detection within each pipe segment, and (4) shape extraction through segmentation of each of the found defects.

The contributions of this article include; (i) a novel feature descriptor that, combined with a classifier, allows for robust defect detection, and (ii) the strategy to apply a specific signal deconvolution algorithm to remove the *double through wall effect* from the RFEC signals to allow the use of standard image segmentation algorithms for shape segmentation of defects in a pipeline. In addition, a method that allows to simulate RFEC measurements of a realistic long pipeline in an extremely efficient manner is developed as part of this work.

The article is organized as follows: the proposed framework including the signal deconvolution, the extraction of the B&S joints, the defect detection, and the defect extraction is discussed in Section 2. The results and validation methods are given in Section 3 followed by the discussion in Section 4 regarding the contributions and limitations.

## 2. Proposed Approach

**Figure 2:**
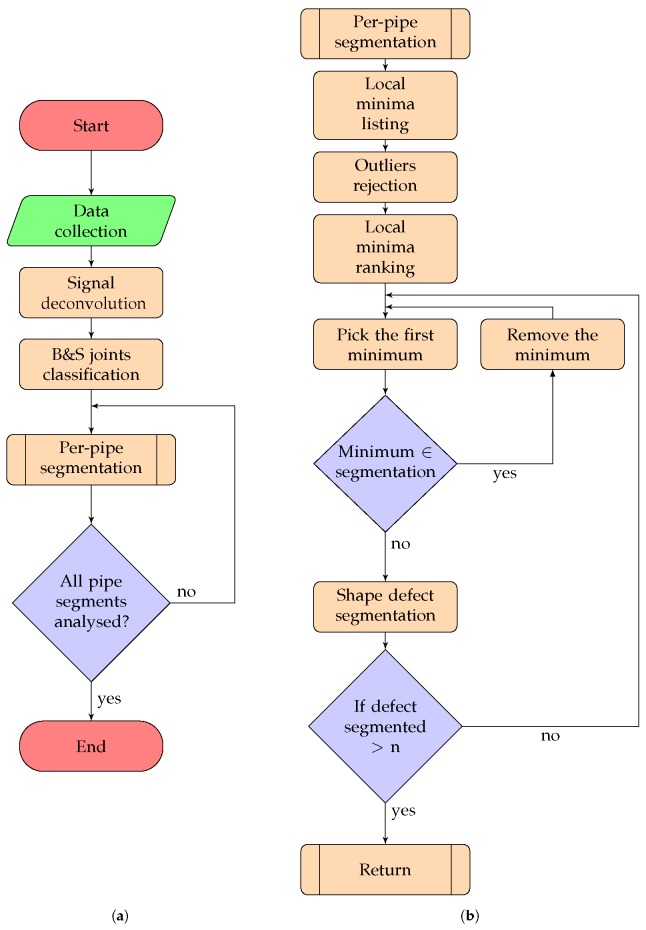
Flowchart of the framework used for the signal segmentation. In (**a**), the flowchart shows the processing done on the complete dataset; in (**b**) the flowchart shows the per-pipe analysis performed for each independent pipe sections.

Automatically extracting the shape of corrosion patches, cracks, and damaged areas of the pipe (referred to with the generic term “defect” in the rest of the paper) from the sensor measurements obtained by an RFEC tool deployed in a pipeline is formulated as a detection and segmentation problem. More formally, the sensor measurements $Y\in R^{X\times \Theta }$ is defined as a 2D matrix with *X* the matrix’s size in the axial direction and Θ in the circumferential direction (the sensor measurements are originally defined in the cylindrical coordinate and are unwrapped into a 2D matrix to make the data processing more convenient). We define $Y_{x,\theta }:=Y\left(x,\theta \right)$ as the sensor measurement located at the position (x,θ) using a cylindrical coordinate system. Given Y, the aim is to find the location $\left(x_{i},\theta_{i}\right)$ and the segmented shape of each of the defects present in the pipeline. The problem is subdivided into multiple independent subproblems. The flowchart of the framework is shown in Figure 2.

### 2.1. Signal Deconvolution

As mentioned above, due to the double through-wall phenomenon, the sensor measurements gathered with an RFEC tool are convolved. In other words, the value of a single sensor measurement is a function of the pipe thickness in several areas. This phenomenon has been widely discussed in the literature [8,11,12]. We proposed to use here the authors’ deconvolution approach described in [10].

The deconvolution in [10] separates the signal Y into two components; (i) the foreground signal $Y^{fg}$ as a 2D matrix and (ii) the background signal $y^{bg}$ that is axisymmetrical over θ and thus represented as a one-dimensional (1D) vector. The relationship between Y, $y^{bg}$, and $Y^{fg}$ is defined by the following equation:

(1) $$Y_{x,\theta }=w_{1}Y_{x,\theta }^{\mathrm{fg}}+w_{k}y_{x}^{\mathrm{bg}},$$

with $w_{1}$ and $w_{k}$ some weight parameters which have to be learned.

The relation described in Equation (1) has been empirically demonstrated using a three-dimensional (3D) FEA simulation in [12]. To perform the signal deconvolution, the background signal $y^{bg}$ needs first to be recovered. This is done by performing a 2D signal deconvolution using the Algorithm 1. Intuitively this algorithm maximizes the similarity between the background signal and foreground signal with a spatial shift equivalent to the tool length. This maximizes the attenuation due to the first crossing near the exciter coil and the attenuation due to the second crossing of the pipe wall near the receiver coils. In fact, for a spatial shift equal to the length of the tool, this attenuation is supposed to be equal. Once the background signal is recovered, $y^{\mathrm{bg}}$ is finally obtained by substituting the recovered $y^{\mathrm{bg}}$ into Equation (1) as

(2) $$Y_{x,\theta }^{\mathrm{fg}}=\frac{1}{w_{1}}\left(Y_{x,\theta }-w_{k}y_{x}^{\mathrm{bg}}\right).$$

$Y^{\mathrm{fg}}$ is then the estimated deconvolved signal, which for the sake of simplicity is re-defined as Y for the remainder of the paper.

The parameters used in Algorithm 1 are defined as follows: Let $l_{exciter-receiver}$ be the distance between the exciter coil and the receivers. δ is defined as the number of measurements recorded by the tool while moving through a distance equal to $l_{exciter-receiver}$. As defined beforehand, *X* is the size of Y in the axial direction. γ is the step size assigned to the gradient descent (line 5). The smoothing part of the algorithm (line 6) is performed by using a simple moving average algorithm.
**Algorithm 1:** Signal deconvolution
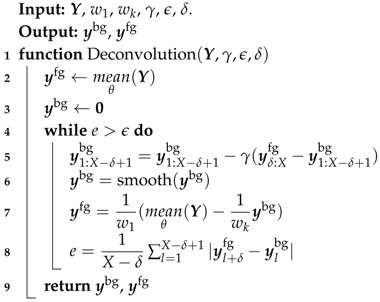


### 2.2. Localization of Bell and Spigot Joints

The full pipeline contains B&S joints that make the defect analysis harder if considered all together with the pipe segments (a pipe segment is defined as a section of a pipeline between two joints). Therefore, once the signal has been deconvolved, each pipe segment is analyzed independently to detect and segment the defects. This approach is referred as per-pipe segmentation in Figure 2. Hence, before considering the segmentation of the sensor data, the pipe joints need to be located within the data. To do so, a similar approach to the one proposed by Vidal-Calleja et al. [13] is used.

#### 2.2.1. Feature Construction

As the B&S joints have an axisymmetrical geometry, the average of the sensor measurements over the circumferential direction is thus considered. More formally, from the sensor measurement Y, a vector y is created, where each $y_{x}$ is defined as $\underset{\theta }{mean}\left(Y_{x,\theta }\right)$. From this vector, an instance—in the machine learning sense—is generated for each measurement and is then classified as either a B&S joint or a non-B&S joint. To do so, a feature vector f is defined for each instance that describes a sensor measurement and these feature vectors are feed into a supervised binary classifier. More formally, the feature vector of the *x*th measurement is defined as follows:

(3) $$f\left(x\right)=[log\left(\bar{B}\right),\bar{\varphi },A,M,C],$$

with $\bar{B}$ the amplitude, and $\bar{\varphi }$ the phase-shift of the circumferential mean value of the sensor measurements. Given that the thickness of the pipe is larger at the joints and that these features are both linearly related to the thickness of the pipe, these particular features provide valuable information on possible joints. [A,M,C] are the Hjorth parameters [14] with *A* the Activity, *M* the Mobility, and *C* the complexity. The Hjorth parameters are time-dependent features created for the description of Electroencephalogram (EEG) signals and are used to describe the behavior of a signal window. These features are defined using an array of phase-shift measurements $\bar{\varphi }$ as follows:

(4) $$\mathrm{Activity}:\;A=\mathrm{var}(\bar{\varphi }).$$

(5) $$\mathrm{Mobility}:\;M=\sqrt{\frac{\mathrm{var}(\bar{\varphi }\frac{d\bar{\varphi }}{dx})}{\mathrm{var}(\bar{\varphi })}}.$$

(6) $$\mathrm{Complexity}:\;C=\frac{M(\bar{\varphi }\frac{d\bar{\varphi }}{dx})}{M(\bar{\varphi })}.$$

As the phase-shift is wrapped over 2π, when the tool passes through outstanding pipe thickness—either smaller or larger than the rest of the pipe—the Hjorth parameters are particularly useful to describe the noisy behavior of the signal in these areas.

Before feeding the data to the classifiers, a soft data whitening on the feature space is performed. Data whitening is particularly important for some of the classifiers as it allows considering each feature with equal importance. With the set of the feature vectors for all instances defined as F, the whitening of the feature space is performed as a standardization method that removes the mean and divides it by the standard deviation.

#### 2.2.2. Classifiers Description

The set of features is then used to train and test a range of different supervised classifiers. The core concept and the main applications for each classifier used herein are summarized below—for all formulations the notation f is used for the feature vector, w for the parameters to be learned, and c for the classes is used:

- *Naive Bayes Classifiers* require the features to be independent and identically distributed. First, a likelihood table is generated for any event by doing a frequency analysis on the training set. A probability of a class $c_{i}$ is then obtained by using the Bayes theorem as $p\left(c_{i}|f\right)=\frac{p\left(c_{i}\right)\prod p\left(f_{j}|c_{i}\right)}{p(f)}$. The class with the maximum probability is then chosen. Naive Bayes classifiers are popular for text classification (e.g., spam filters) and have also been applied for medical diagnosis [15]. It is possible to train the classifier with a closed-form expression [16], which allows training with a linear computational complexity.
- *Logistic Regression* uses a logistic function—also known as a sigmoid function—defined as $p\left(f\right)=\frac{1}{1+e^{w^{T}f}}$ to generate a probability. A threshold on the probability is then used to yield a binary classification [17]. Logistic regression is used in many fields, such as medical [18,19] and social sciences [20].
- *Random Forests* are obtained by training a set of independent decision trees on a set of randomly sampled data. Once these decision trees are trained, a new sample is classified by considering the class which is most often obtained from all the independent decision trees [21]. The utilization of multiple classifiers is referred to as bagging and is used to avoid overfitting [22].
- *Support Vector Machine* finds the linear decision boundary which maximizes the distance between the closest points of each class—i.e., finds a fat margin—while minimizing the distance between the miss-classified samples and the decision boundary [23]. Additionally, to create a more flexible classification, a *kernel trick* [24] can be used to bring the features into a higher dimension (in our case the Support Vector Machine (SVM) is using a Radial Basis Functions (RBF) kernel).

A study comparing the performance of 179 different classifiers tested against 121 datasets has been done by Fernández-Delgado et al. [25]. The study shows that *boosting algorithms*, *SVM* and particularly *Random Forests* are most often outperforming other classifiers, with *Random Forests* often ranked amongst the three best classifiers. Additionally to these state-of-the-art classifiers, we have chosen *Logistic Regression* and *Naive Bayes Classifiers* for their low complexity which often lead to a better generalization.

Once the position of every joint is located, it is possible to analyze each pipe segment independently. Thus, in the following sections, Y is used to describe the 2D sensor measurements for a single pipe segment.

### 2.3. Defect Detection

Many segmentation algorithms require either a seed or a Region of Interest (ROI) as an initialization step. Therefore, detecting defects is a required task before performing the segmentation. In this context, the *defect detection* is defined as the task of finding the minimum thickness point of either a corrosion patch, a crack or a localized pitting. The defect detection is performed by ranking the local minima within the sensor measurements. The outliers amongst the potential defects are then discarded with a binary classifier. Most outliers are local minima near the edges of the pipe segment (i.e., near the joints).

First, to find the local minima, a peak finder algorithm that performs a comparison between the considered pixel and its neighbors using an 8-connectivity window is used. More formally, a sensor measurement $Y_{x,\theta }$ is considered to be a local minimum if it respects the following condition:

(7) $$Y_{x,\theta }=\min\left[\begin{array}{ccc} Y_{x-1,\theta -1} & Y_{x-1,\theta } & Y_{x-1,\theta +1}\\ Y_{x,\theta -1} & Y_{x,\theta } & Y_{x,\theta +1}\\ Y_{x+1,\theta -1} & Y_{x+1,\theta } & Y_{x+1,\theta +1} \end{array}\right].$$

**Figure 3:**
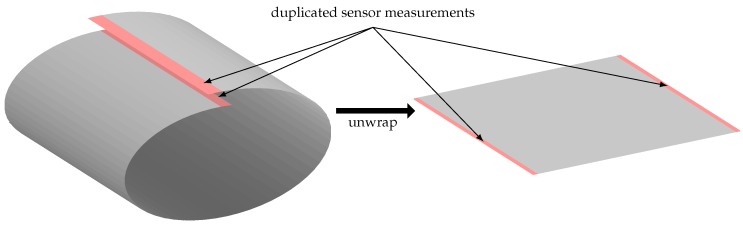
While unwrapping the data, the sensor measurements located at the boundary regarding theta are duplicated on the other extremity of the data, to consider the cylindrical properties of the sensor measurement.

As the connectivity of each measurement is used to find the potential defects, the sensor measurement located on the edges of the 2D sensor measurements are not considered properly—in other words, it is not possible to define an 8-connectivity for these points. To overcome this problem, the cylindrical property of the sensor measurement is used to wrap the data on the circumference as shown in Figure 3. More formally, the lines of measurements located at the extremity on θ—shown in red on the figure— are duplicated on the other extremity of the 2D image while unwrapping the sensor measurement.

The search of all the local minima can then be implemented efficiently by using a divide-and-conquer strategy with an O(n) complexity [26]. Efficient implementations of this algorithm are common within popular libraries and often leverage Graphics Processing Units (GPU) to reduce the computation time (*scipy.ndimage.filters.minimum_filter* for Python or *imregionalmin* for Matlab implementations). The list of local minima is then ranked according to the value of the phase-shift, and the outliers from the list are removed using a binary classifier. The new feature vector f used to train the classifier is defined as follows:

(8) $$f=[log\left(B\right),\varphi ,A,M,C,d^{\mathrm{upstream}},d^{\mathrm{downstream}}],$$

with *B* the amplitude, φ the phase-shift, and [A,M,C] defined similarly to Section 2.2.1—conversely to the previous section, these features are not defined based on the circumferential mean value. The additional features $[d^{\mathrm{upstream}},$ $d^{\mathrm{downstream}}]$ are the distance to the joints upstream and downstream. Due to the large thickness of the joints, the strength of the magnetic field is weak, leading to a low signal to noise ratio. In brief, the distances to the joints upstream and downstream describe how close the points are from a source of noise.

The rejection of the outliers is then performed by classifying every potential defect based on the features vector f. We compare the same classifiers described in Section 2.2.2 for this task.

### 2.4. Defect Segmentation

As discussed in the introduction, many algorithms are available for the segmentation task. Considering the limited size of our datasets, we focus here on non-supervised methods. We have chosen for our analysis the principle of the *region growing* algorithm and the *active contour* segmentation. The former one is chosen as it has shown good performance for electromagnetic data, and the latter one is chosen for its increased flexibility compared to *region growing*.

#### 2.4.1. Region Growing

For a given 2D image $Y\in R^{X\times \Theta }$, an initial seed $s\in N^{2}$ and a threshold parameter t∈R, the *region growing* algorithm starts the segmentation of Y by allocating *s* to the segmented area O. An iterative process is then started by comparing the value of each unallocated neighbor of O to the mean value of the pixels in O. If this measure is below the threshold *t*, then the neighbor is allocated to O and the comparison/allocation of the neighbors is performed again. More formally, the region growing algorithm is defined by Algorithm 2.

The seed is obtained from defect detection described in Section 2.3. Thus, the threshold *t*—which is bounded by 0 and the range of Y—is the only parameter that has to be optimized. For these reasons, a simple way to optimize *t* is to perform an exhaustive search by using a grid search over the possible values of the parameter *t* and choose the value with the best performance.
**Algorithm 2:** Region Growing
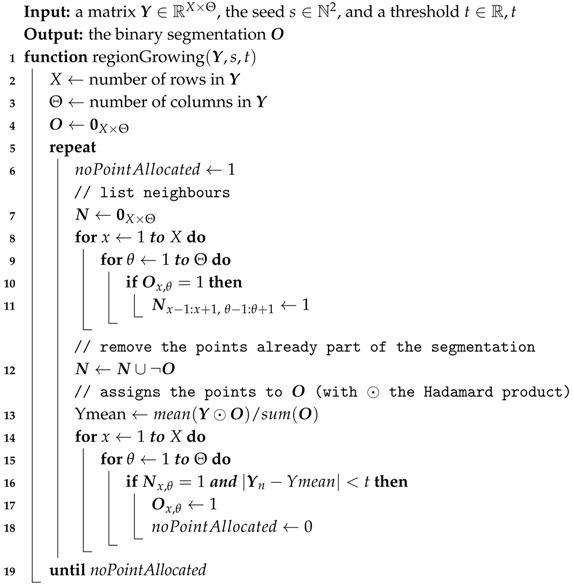


#### 2.4.2. Active Contour Segmentation

The *active contour* algorithm is an iterative method which finds the segmentation contour that minimizes an energy function *F*. While several variants of the active contour algorithm exist, the core of the energy function is often defined as the sum of the dissimilarity of the pixels inside the contour and the dissimilarity of the pixels outside the contour. We focus here on the method from Chan and Vese [27], called *Active Contour Without Edge*, where additional regularization terms are added to the energy function *F* to constrain the length of the contour and the surface area of the inner segmentation. These regularization terms allow smoother segmentations. The energy function *F* is then defined as follows

(9) $$\begin{array}{cc} F(c_{1},c_{2},C) & =\mu \mathrm{Length}(C)+\nu \mathrm{Area}(inside(C))\\ & +\lambda_{1}\int_{inside(C)}|Y_{x,\theta }-c_{1}{|}^{2}dxd\theta +\lambda_{2}\int_{outside(C)}{\left|Y_{x,\theta }-c_{2}\right|}^{2}dxd\theta , \end{array}$$

where *C* is the contour to optimize, $c_{1}$ is the average value of the image inside of *C*, and $c_{2}$ is the average value of the image outside of *C*. μ and ν are regularization parameters that restrict the length and the area of the contour. $\lambda_{1}$ and $\lambda_{2}$ are the parameters that weight the importance of the similarity between the inside and outside of the contour. In practice, the minimization of the energy *F* is solved as a level set formulation where a Lipschitz function ϕ:Ω→R is used as a proxy instead of manipulating the contour *C*. With the given ϕ function, the contour is then defined as

(10) $$\left\{\begin{array}{c} C=\{(x,\theta )\in \Omega :\phi (x,\theta )=0\},\\ inside(C)=\{(x,\theta )\in \Omega :\phi (x,\theta )>0\},\\ outside(C)=\{(x,\theta )\in \Omega :\phi (x,\theta )<0\}, \end{array}\right.$$

and the terms from Equation (9) are then defined as follows:

(11) $$\mathrm{Length}\left(C\right)=\int_{\Omega }\left|\nabla H\left(\phi \left(x,\theta \right)\right)\right|dxd\theta ,$$

(12) $$\mathrm{Area}\left(inside\left(C\right)\right)=\int_{\Omega }H\left(\phi \left(x,\theta \right)\right)dxd\theta ,$$

(13) $$\int_{inside(C)}|Y_{x,\theta }-c_{1}{|}^{2}dxd\theta =\int_{\Omega }\left|Y_{x,\theta }-c_{1}\right|H{\left(\phi \left(x,\theta \right)\right)}^{2}dxd\theta ,$$

(14) $$\int_{outside(C)}|Y_{x,\theta }-c_{2}{|}^{2}dxd\theta =\int_{\Omega }\left|Y_{x,\theta }-c_{2}\right|{\left(1-H\left(\phi \left(x,\theta \right)\right)\right)}^{2}dxd\theta ,$$

with H(.) the Heaviside function, and $c_{1}$ and $c_{2}$ defined as:

(15) $$\begin{array}{cc} c_{1}\left(\phi \right) & =\frac{\int_{\Omega }Y_{x,\theta }H\left(\phi \left(x,\theta \right)\right)dxd\theta }{\int_{\Omega }H\left(\phi \left(x,\theta \right)\right)dxd\theta },\\ c_{2}\left(\phi \right) & =\frac{\int_{\Omega }Y_{x,\theta }\left(1-H\left(\phi \left(x,\theta \right)\right)\right)dxd\theta }{\int_{\Omega }\left(1-H\left(\phi \left(x,\theta \right)\right)\right)dxd\theta }. \end{array}$$

The evolution of the ϕ surface is obtained by solving the following Partial Differential Equations (PDE) (the time *t* is a virtual variable introduced for solving the PDE equation. It has no physical meaning related to the problem):

(16) $$\begin{array}{c} \frac{\partial \phi }{\partial t}=\delta_{\epsilon }\left(\phi \right)\left[\mu \;\mathrm{div}\left(\frac{\Delta \phi }{\left|\Delta \phi \right|}\right)-\nu -\lambda_{1}{\left(\mu_{0}-c_{1}\right)}^{2}+\lambda_{2}{\left(\mu_{0}-c_{2}\right)}^{2}\right]=0\;\mathrm{in}\;\left(0,\infty \right)\times \Omega ,\\ \phi \left(0,x,\theta \right)=\phi_{0}\left(x,\theta \right)\;\mathrm{in}\;\Omega ,\\ \frac{\delta_{\epsilon }\left(\phi \right)}{\left|\Delta \phi \right|}\frac{\partial \phi }{\partial n}=0\;\mathrm{on}\;\partial \Omega , \end{array}$$

with n the exterior normal of the contour *C*, $\frac{\partial \phi }{\partial n}$ the normal derivative of ϕ at the contour *C*, and $\delta_{\epsilon }$ the regularized Dirac function. The formal steps required for solving the *active contour without edge* are given in Algorithm 3. The re-initialization described in line 6 is required to avoid the level set function from becoming too flat.

**Remark (cylindrical space):** The active contour algorithm is designed for 2D matrices. As a result, the regularization term Equation (11) is integrated over the matrix edges. For the cylindrical RFEC data, this problem is overcome by rotating the matrix to be centered on the defect that has to be segmented and then applying the inverse transformation on the segmented data.

The parameters $\lambda_{1}$, $\lambda_{2}$, μ, and ν have to be tuned for adapting the methodology to the RFEC dataset. Conversely to the Region Growing approach, performing an exhaustive search on such a parameter space is not feasible. Thus, we propose to search for the best parameters using Genetic Algorithm (GA) which aims for a globally optimal solution [28].
**Algorithm 3:** Active Contour Without Edges
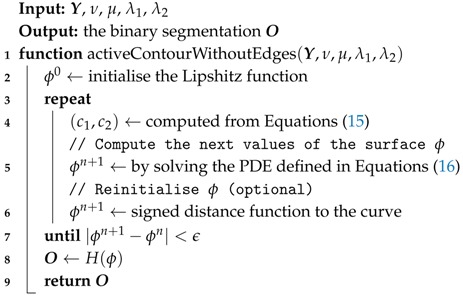


## 3. Results

Two datasets are used for the evaluation and validation of the proposed framework. The first dataset consists of RFEC sensor measurements artificially obtained using a calibrated direct model applied over a realistically corroded pipe geometry generated by spatial statistics. This controlled dataset allows us to generate a large amount of high quality data to evaluate the individual algorithms. The second dataset consists of RFEC sensor measurements obtained with a commercial tool and a limited amount of ground truth obtained by excavating, cleaning and laser scanning corroded pipe sections from the assessed pipeline. Note that this latter dataset is limited due to the high cost of data collection, not only regarding the inspection but regarding the excavated pipes.

### 3.1. Artificial Dataset

**Figure 4:**
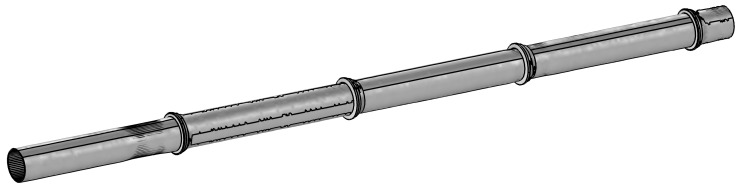
3D visualization of a part of the artificial pipeline. The artificial pipe segments are linked to each other using the 3D model of a Bell and Spigot joint.

The geometry of the pipe segments that captures the distribution of the corrosion can be learned with spatial statistics and data-driven approaches [29]. Such models are trained on the real thickness data obtained from excavated pipe segments, giving the possibility to generate plausible corroded geometries. Using the model proposed by Shi et al. [29], a set of realistic looking pipe segments has been generated. Each pipe segment has to be linked to each other using joints in the same way that real pipelines are laid. The 3D profile of a B&S joint, previously scanned with a laser scanner and later digitalized has been inter-placed between each artificial pipe segment. This results in an artificial pipeline made of realistically corroded 3D pipes. A sample of this pipeline is shown in Figure 4.

The following step consists of generating the associated sensor signal, i.e., the simulated inspection of the artificial pipeline by an RFEC tool. We use a previously characterised direct model to map the geometry into sensor measurements [8,10]. The parameters used for the direct model are obtained from the 2D axisymmetric FEA simulation; therefore, applying it to a 3D case results in an approximation. Still, this process allows for the production of a realistic dataset which can be used to test the proposed framework.

Using the formalism developed in [10] and starting from Equation (1) with the cylindrical coordinates:

(17) $$Y=w_{1}T^{\mathrm{fg}}+w_{k}t^{\mathrm{bg}}+y_{0}+N\left(0,\sigma^{2}\right),$$

where Y is the sensor measurements. In our simulated data $w_{1}=-158.33$, $w_{k}=-115.08$, and $y_{0}=65.89$ are the parameters from the direct model (in the case of a real system, these parameters would depend on the properties of the tool). $N(0,\sigma^{2})$ is the sensor noise defined as Gaussian noise with zero mean and σ for standard deviation. $T^{\mathrm{fg}}$ is the thickness map from the pipeline and $t^{\mathrm{bg}}$ is a vector defined by the circumferential thickness average such that

(18) $$t^{\mathrm{bg}}\left(x\right)=\frac{1}{2\pi }\int_{\theta =0}^{2\pi }T\left(x+k-1,\theta \right).$$

Similarly to the inverse model proposed in [10], $T^{\mathrm{fg}}$ and $t^{\mathrm{bg}}$ are shifted by the length of the tool in term of indices, i.e., $T^{\mathrm{fg}}=T\left(1:n-k,1:\Theta \right)$ and $t^{\mathrm{bg}}=t\left(k:n\right)$.

The outcome of this process is a new dataset which has both the sensor measurements and the thickness map—used to produce the ground truth for validating the algorithms.

### 3.2. Real Dataset

**Figure 5:**
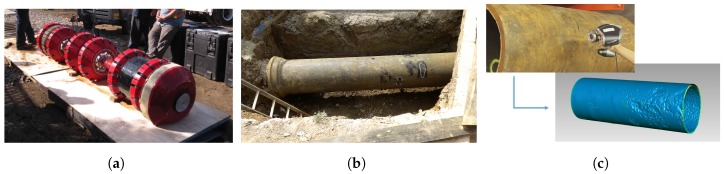
(**a**) Commercial RFEC tool used for the field data collection in this work, courtesy of PICA Corporation, Edmonton, AB, Canada; (**b**) Pictures of an excavated pipe section with the Bell and Spigot joint visible on the left; (**c**) Digitalization of a 3D model of the pipe using a laser scanner. The 3D model is then transformed into a 2.5D thickness map.

Actual data from a condition assessment inspection with the See-Snake RFT tool (Figure 5a) is also used to evaluate and validate the results of the proposed framework. Approximately 800 m of data have been inspected on a 660 mm diameter cast-iron water pipe. Ground-truth for the joints has been manually obtained by analysts from PICA Corporation. and, for the real thickness of the pipe, a few selected pipes have been excavated and scanned with a high resolution 3D laser scanner and high resolution accurate thickness has been obtained as described by Skinner et al. [30] (see Figure 5b,c).

The sensor measurements Y are obtained by scanning the entire pipeline. The tool is composed of a single exciter coil, an array of electromagnetic receivers distributed along the circumferential direction. The tool is pushed downstream in the pipeline by the water flow. While traveling down the pipe, the tool generates an electromagnetic field and records the response from the interaction between the electromagnetic field and the ferromagnetic pipe. The measurements recorded by the tool are the amplitude and the phase-shift—described in Section 1—from the array of receivers. Prior to the signal deconvolution step, the collected data is pre-processed to interpolate missing data and re-calibrate receivers [31].

### 3.3. Classifiers Evaluation

As the proposed framework relies heavily on classification algorithms, we have dedicated here a full section to evaluate with a rigorous methodology the classifiers described in Section 2.2.2 to assure accurate segmentation results. Let us consider a set of *N* input-output $D={\left\{\left(f_{i},l_{i}\right)\right\}}_{i=1}^{N}$—called training data—where $f_{i}\in F$ is a *d*-dimensional feature vector and $l_{i}\in L$ is the class where *L* is defined as {true,false}. A binary classifier learns a function $\hat{h}$ which tries to approximate the function h:F→L.

#### 3.3.1. K-Fold Cross-Validation

During the training stage of a classifier, it is possible to have the classifier over-fitting the training data. This problem mostly occurs when the classifier’s structure has a high complexity and the dataset is relatively small. In this case, the classifier tends to memorize the training data instead of learning the correlation between the features and the label. A common method used to avoid being over-confident in the classification performance is to use a *K-fold cross-validation*. It consists of splitting the training dataset into *K* sets and using one of these sets for evaluating the classifier while the other sets are used for training. This operation is repeated *K* times changing the evaluation set each time. Unless another method is specified in a subsection, the classifier’s parameters are trained by using a 10-fold cross-validation.

#### 3.3.2. Confusion Matrix and Standard Metrics

The performance of the binary classifier can be represented in a table called *confusion matrix* by summarizing correctly and incorrectly classified instances according to their respective classes. The categories are named as follows: true positives tp, false positives fp, true negatives tn, and false negatives fn. These categories are defined according to the confusion matrix shown in Table 1. A confusion matrix provides the raw information about the classification success. However, when optimizing an algorithm, it is often more practical to use a more compact representation of the classifier success. Following this idea, several measures are defined to evaluate the performance of a classifier such as

$$accuracy=\frac{tp+tn}{tp+tn+fp+fn},precision=\frac{tp}{tp+fp},recall=\frac{tp}{tp+fn},\mathrm{fall-out}=\frac{fp}{fp+tn}\;.$$

Despite the *accuracy* and the *precision* being commonly used for evaluating classifiers, they are a biased representation of the reality in the case where the instances are not distributed evenly amongst the classes—referred to as unbalanced datasets. In such a situation, the *Cohen’s kappa coefficient* [32] can be used as an alternative to the standard *accuracy* measure as it compares the observed accuracy to the expected accuracy of a random classifier. It is formally defined as follows,

(19) $$\kappa =1-\frac{1-p_{o}}{1-p_{e}},$$

with $p_{o}$ the probability of an instance to be correctly classified and $p_{e}$ the probability of an instance to be correctly classified using a random classifier. More formally $p_{o}$ and $p_{e}$ are defined as follows for a binary classifier,

$$p_{o}=\frac{tp+tn}{N},p_{e}=\frac{\left(tp+fp\right)\cdot \left(tp+fn\right)+\left(fn_{t}n\right)\cdot \left(fp+tn\right)}{N^{2}}.$$

#### 3.3.3. ROC Curves

Considering a class *c* defined within {1,…,C}, for a given instance $\left(f_{i},l_{i}\right)$, most classifiers produce a set of probabilistic predictions $\forall c,p(l_{i}=c|f_{i})$ and the most likely class is defined by $\hat{l_{i}}=\overset{C}{\underset{c=1}{\mathrm{argmax}}}\;p\left(l_{i}=c|f_{i}\right)$. In the case of a binary classifier, where the class is reduced to {true,false}, the probabilities of each class have the following property: $p\left(l_{i}=true|f_{i}\right)=1-p\left(l_{i}=false|f_{i}\right)$. Thus, the classification task is performed by choosing a threshold which decides, for a given probability, to which class the instance belongs—without prior information, one would choose the threshold 0.5.

**Figure 6:**
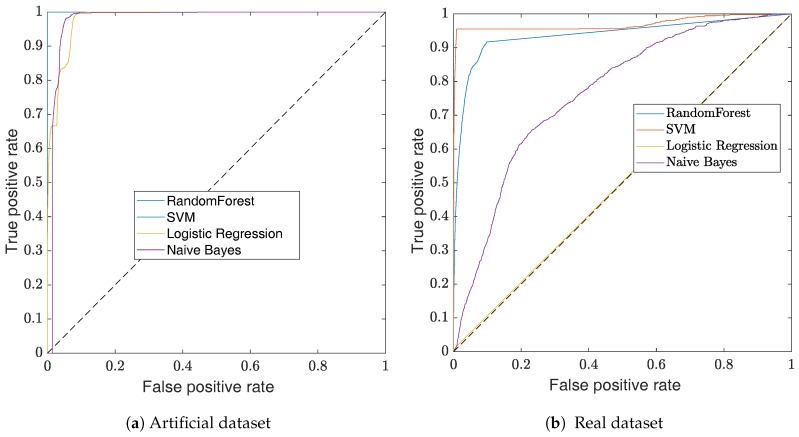
ROC curves of the B&S classification. In (**a**) the ROC curves show the performance of the classifiers for the artificial dataset and in (**b**) the ROC curves show the performance of the classifiers on the real dataset. Overall, for both dataset, the SVM classifier outperforms the other classifiers. In (**a**), the SVM and the random forest classifiers have the same performance.

The impact of the threshold choice can be visualized with Receiver Operating Characteristic (ROC) curves. This visualization method, illustrated in Figure 6, is a graphical plot that illustrates the performance of a binary classifier for different discrimination thresholds. Practically, the curves are created by plotting the *recall* against the *fall-out* while varying the value of the classifier threshold. The use of the *recall* and the *fall-out* to define the ROC curve makes it insensitive to unbalanced datasets [33]. The ROC curve of a perfect classifier would have the shape of a step function, where a random classifier would be a straight line—shown as a dotted line in Figure 6.

### 3.4. Signal Deconvolution

**Figure 7:**
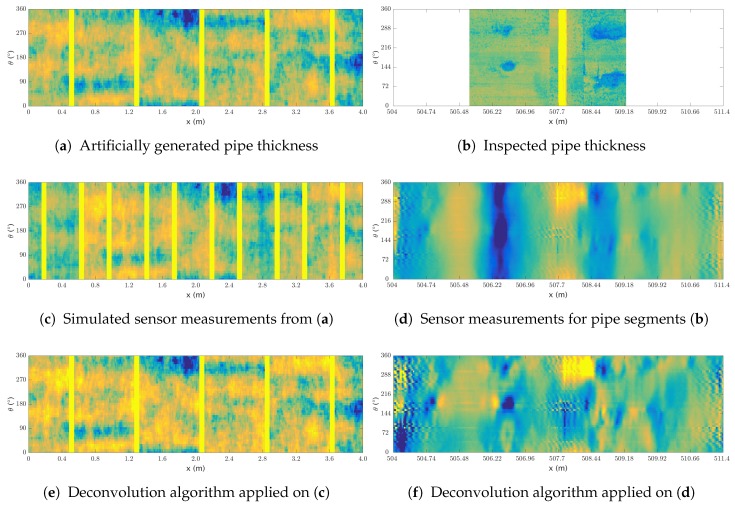
Samples from the signal deconvolution applied to the artificial (left column) and real (right column) dataset. The pipe profile is shown in the first row, the associated RFEC signal is shown in the second row, and the applied signal deconvolution is shown in the last row. For both dataset, darker colors relate to lower thicknesses—the yellow stripes correspond to joints.

The signal deconvolution is applied to both the artificial and real dataset using the methodology described is Section 2.1. For the simulated dataset, $w_{1}$ and $w_{k}$ were learned from the FEA and found empirically for the real dataset, ϵ was set to $10^{-3}$ and γ to 0.1. For each dataset, a sample of the deconvolution result is shown in Figure 7, with the artificial dataset in the first column and the real dataset in the second one. The pipe profile for each dataset is shown in Figure 7a,b, and the associated RFEC data in Figure 7c,d. The outcome of the signal deconvolution is shown in Figure 7e,f.

As described in Section 2.1 and Section 3.1, the algorithm used for the signal deconvolution is the inverse of the function used to simulate the artificial measurements (with additional Gaussian noise). Thus, the correct performance of the signal deconvolution shown in Figure 7e is expected. Conversely to the artificial dataset, in the real scenario, the magnetic field generated by the exciter coil flows trough the path of least resistance. Therefore, the applied signal deconvolution—which assumes the magnetic field to flow through a constant path—is an approximation of the reality. In order to attenuate the effect of this approximation, a smoothing step is used in Algorithm 1 (line 6). In the real data sample, the filtering effect of the deconvolution is visible in Figure 7d where the circumferential offset at the axial position 506.5 m is removed after the deconvolution—as shown in Figure 7f. As a result, the two large defects visible in the pipe profile Figure 7b are highlighted after the signal deconvolution.

### 3.5. Bell and Spigot Joint Detection

In this section, we use the methods described in Section 3.3 to evaluate the B&S joint classifier. At first, we consider the ROC curves shown in Figure 6 to compare the classifiers’ performances.

For each dataset, both the SVM classifier with an RBF kernel and the *random forest* classifier show good performances. The poor performance of the logistic regression classifier on the real dataset is due to the unbalance dataset and the definition of its loss-function, which maximizes the global accuracy. As a result, all the instances are classified in the same class. While SVM slightly outperforms random forest for any threshold, it has a training time complexity of $O(N^{2})$ [34]. The time complexity is given according to the training dataset that has *N* instances each with *d* features. This complexity is due to the RBF kernel, which provides a more powerful classification compared to the linear kernel. In practical scenarios, the linear kernel often performs similarly to the logistic regression. Due to the RBF kernel, SVM is slower than random forest which has a complexity of O(dNlog(N)) [35]. In our case, all computations are performed off-line. Thus, the SVM classifier with an RBF kernel is preferred. For online implementation, one might consider a different classifier to reduce the computation time. More importantly, the good performances of all selected classifiers show that the features used for the classification contain the information required for the detection of B&S joints.

While it is common to classify the instances by using a threshold of 0.5 on the output probability, in a practical scenario, one has to consider if false positives are preferred to false negatives—or vice versa. Here, as we classify the sensor measurements $y_{i}$, there are multiple instances which are associated with a single B&S joint (around 15 instances for a single joint). Thus, the presence of a few false negatives is not critical as we just need to detect at least one instance for each joint. On the other hand, the false positives result in interpreting an element of a pipe segment as a B&S joint, which does not allow a per-pipe analysis. Considering the ROC curve, the threshold is set to 0.5 for the artificial dataset which results in a perfect classification—as shown in Table 2. For the real dataset, a threshold of 0.7 has been chosen as this value allows having a classification without false positives. The performance of the classifiers for the chosen threshold is displayed in Table 3. Despite the fact that we are focused on the Precision measure, this dataset is a good example of why the *accuracy* should not be used to measure the performance of unbalanced datasets.

**Figure 8:**
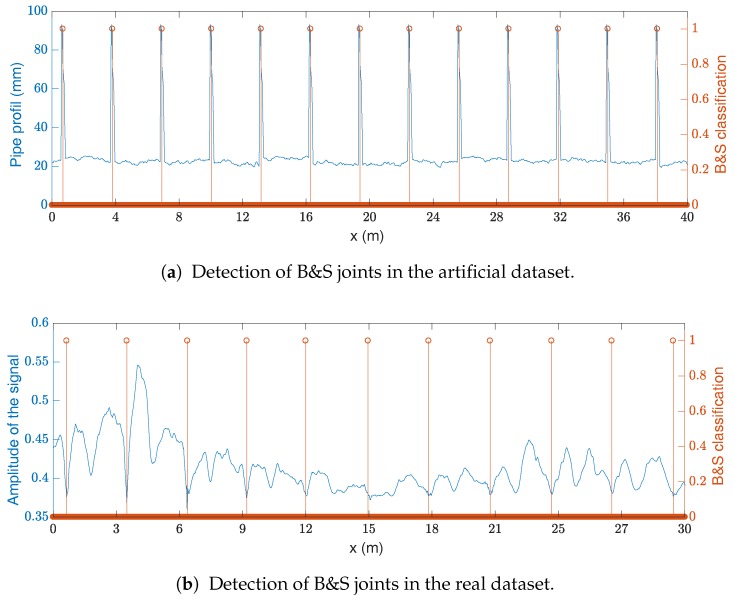
B&S classification. In (**a**), the geometry of the artificial pipe is shown with the B&S classification; In (**b**), the signal is shown with the B&S joint for this dataset.

The visualization of the classification performance is shown in Figure 8. A sample of the artificial pipe profile—in blue—and the B&S joint classification—in orange—are shown in Figure 8a. For the real dataset, as the pipe profile is not available for a large section, the B&S joint classification is compared with the sensor measurements—in blue—in Figure 8b. In Figure 8b, the success of the classification is shown by the regularity of the classification (i.e., the B&S joints are regularly spaced by a pipe segment length).

### 3.6. Defect Detection

This section evaluates the performance of the different classifiers for detecting the center of a defect as described in Section 2.3. In the artificial dataset, the only source of noise comes from the simulated Gaussian noise $N(0,\sigma^{2})$ on the signal. There are no outliers present in the data, hence there is no need to train a classifier here. Therefore, in this subsection we only analyze the results obtained on the real dataset.

Since we are using supervised learning, ground truth is needed not only for validation but also for training the algorithms. For this purpose, a dataset of 1000+ samples has been manually labeled. The collected dataset contains 711 instances labeled as outliers and 359 minima labeled as defects.

**Figure 9:**
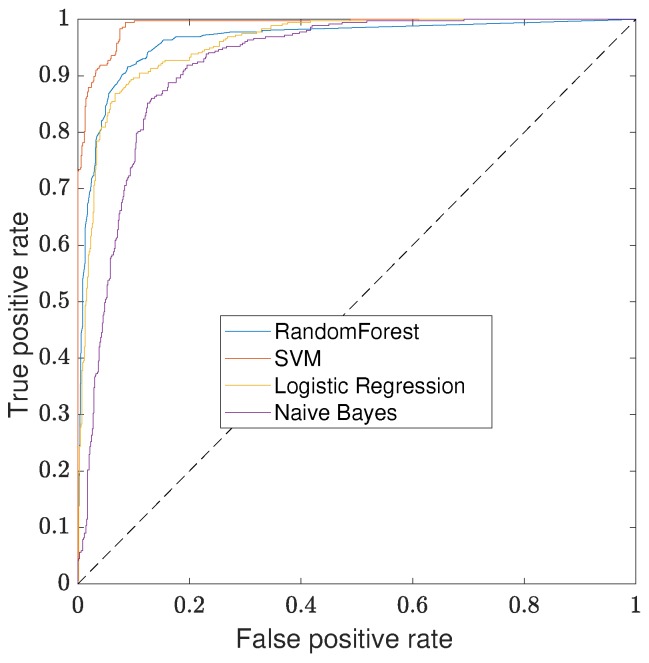
ROC curves of defect classification in real dataset comparing the different classifiers. SVM with an RBF kernel outperforms the other classifiers.

Once again, an ROC curve, shown in Figure 9, is used for comparing the performance of the different classifiers. Similarly to the B&S joint classification, the SVM classifier outperforms the other methods. However, in this case, the size of the dataset is small enough not to have to worry about the computation time. Considering the engineering problem and the overall performance of the classifier, there is no preference for balancing the number of false positives versus the false negatives. As a result, the threshold for the classifier is set at 0.5 on the output probability. The performance on a 10-cross fold-validation of each classifier for this threshold is given in Table 4.

Additionally, the confusion matrix summarizing the SVM classification for the given threshold is given in Table 5. The performance of the classifier is acceptable considering that some of the points were manually labeled as outliers where they could have arguably been considered as the center of a defect.

### 3.7. Defect Segmentation

**Figure 10:**
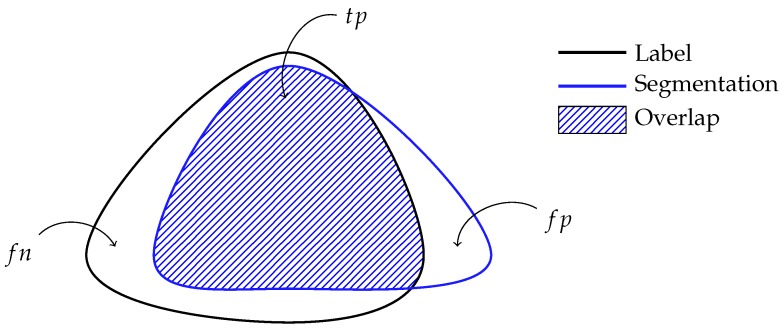
Relation between segmentation and classification evaluation. For a label *L* and a segmentation *S*, the true positives are defined as the intersection of the label and segmentation or tp=L∩S, the false positives consist of the segmentation absent in the label or fp=S\L, the false negatives are the label absent in the segmentation or fp=L\S , and the true negatives are the pixels absent in both the segmentation and label or tn=ω−(fp+tp+fn).

This section evaluates the performance of the different segmentation algorithms described in Section 2.4. While performing fundamentally different tasks, segmentation and classification algorithms can be evaluated similarly [36]. To do so, each pixel is considered to be an instance of the dataset. The notion of true/false positives and true/false negatives are then defined similarly to the example shown in Figure 10.

In the segmentation context, the *precision* relates to the proportions of correctly segmented pixels within the segmentation, and the *recall* relates to the proportions of correctly segmented pixels from the label. While these measures provide valuable information for the segmentation performance, segmentation algorithms are usually evaluated using the *F*-score [37], which is often referred to as the harmonic mean and is defined as follows:

(20) $$\mathrm{F-}\mathrm{score}=\frac{2\times recall\times precision}{recall+precision}.$$

The evaluation criteria *F*-score is used as the reward function which has to be maximized for optimizing the segmentation parameters. For both GA implementations, we use the same hyper-parameters in the optimizer: the probability of crossover is set to 0.8, the probability of mutation to 0.01, the population to 50, and the maximum number of generations to 100. The stability of these parameters has been tested with 40 different initializations of the randomizer resulting in small variations in the *F*-score (2.62% for the generated dataset and 3.09% for the real dataset).

**Figure 11:**
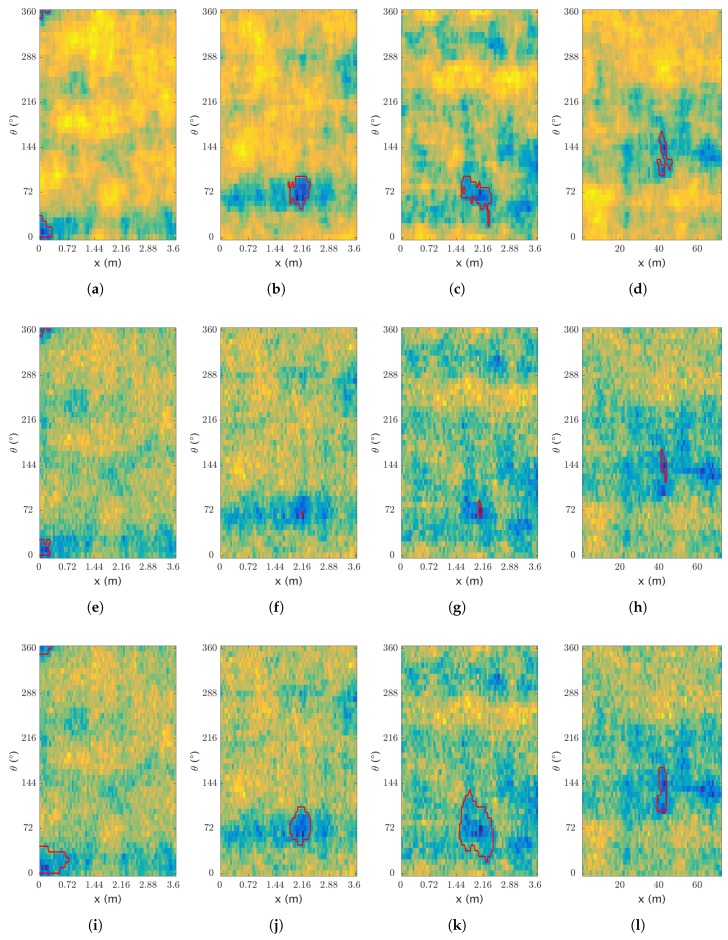
Segmentation on artificial data. Results of the segmentation, with (**a**–**d**) the ground truth and the manual segmentation; (**e**–**h**) and (**i**–**l**) the processed sensor data with (**e**–**h**) the region growing segmentation and (**i**–**l**) the active contour segmentation—darker colors relate to lower thicknesses.

The segmentation label of the artificial dataset has been obtained by applying a threshold on the thickness map of the pipeline. This approach provides an unbiased label and helps to automate the process. We use a 10-fold cross-validation for assessing the results which are presented in Table 6. The *precision*, *recall*, and *accuracy* are also given even though they are not used for optimizing the parameters directly. Additionally, some samples are provided showing the results of the segmentation in Figure 11.

**Figure 12:**
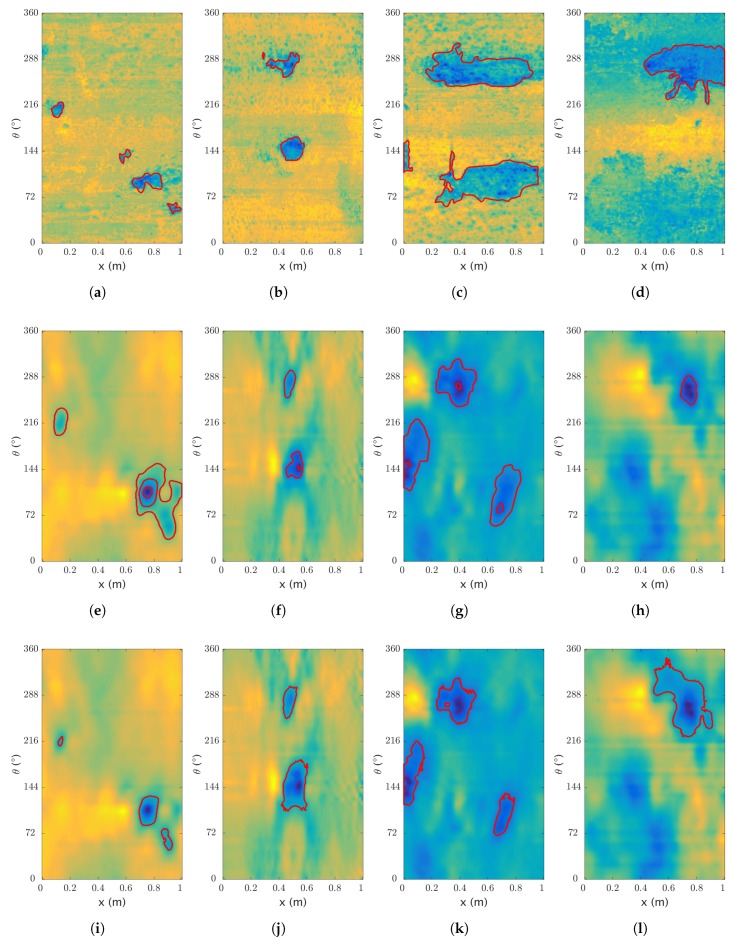
Segmentation on real data. Results of the segmentation, with (**a**–**d**) the ground truth and the manual segmentation; (**e**–**h**) and (**i**–**l**) the processed sensor data with (**e**–**h**) the region growing segmentation and (**i**–**l**) the active contour segmentation—darker colors relate to lower thicknesses.

In the case of the real dataset, the thickness map of the pipe has been manually labeled to generate the segmentation of the reference. This manual label is used to evaluate the performance of the algorithm. Once again, the *F*-score is used as a metric to optimize the parameters. As the data is more limited than the previous dataset, we use here a leave-one-out-cross-validation (a leave-one-out-cross-validation is equivalent to K-fold cross-validation with K equal to the number of instances) while optimizing the parameters on the rest of the dataset. The *precision*, *recall*, *accuracy* and *F*-score are given in Table 7 and results are shown in Figure 12.

## 4. Discussion and Final Remarks

The proposed framework solves the problem of extracting the size and shape of large corrosion patches and other defects from data collected with RFEC tools. The framework considers multiple steps such as signal deconvolution using the methodology proposed in [10], joint and defect detection are solved using an SVM classifier, and defect segmentation using active contour. Results are tested on both artificial and real datasets using standard metrics for evaluation of classification problems and compared to other standard algorithms.

The key contributions within the framework are the proposed embedding of the 2D signal deconvolution into the framework, which allows the use of state-of-the art segmentation algorithms, and the novel feature used to robustly locate defects. This feature is extracted from the minima in the measurements of each independent receiver around the circumference and fed into the classifier to remove potential outliers. Furthermore, a minor contribution of this paper is the proposed mechanism to generate RFEC data to evaluate the algorithms such as detection and segmentation.

In order to understand the importance of this work, one has to compare the proposed work with the work applied to other NDE methods. For instance, RFEC is often compared to PEC technologies for the assessment of ferromagnetic pipeline. Both technologies are based on two coils; an exciter coil and a receiver coil. PEC probes, are often designed as hand devices. Hence they can be used for assessing pipes from the outside. As RFEC tools are most often designed as inline tools, they allow assessing several kilometers of pipe in a single inspection. While RFEC tools offer a more convenient approach in terms of inspection, the data analysis is more complicated. Indeed, solving the inverse modeling of PEC tools can be performed by linear or non-linear regression mapping of the sensor measurements into the thickness map (typically using the decay rate of the PEC measurements [38]). In the case of RFEC tools, the data interpretation is more complicated and the spatial dependency of the sensor measurements has to be accounted for. This problem is solved as described in this paper by the signal deconvolution step which then allows using standard classification and segmentation algorithms afterward. Without the signal deconvolution, using any standard segmentation method to get the defects would result mostly in vertical stripes (see Figure 7d). Thus, the final segmentation shown in the results section could not have been obtained even by manually processing the data.

Considering the vast variety of available segmentation algorithms, one might argue about the choice of the active contour algorithm. More powerful segmentation algorithms, such as superpixels [39] and fully connected CNNs [40], offer higher complexity. However, they are harder to train and more prone to overfitting when not enough data is available. Furthermore, simpler approaches based on edge detection such as Canny, Sobel, or gradient would not perform properly due to the smooth nature of the data. The performances of the active contour segmentation presented in Table 7 are acceptable given the fact that they include all the accumulated errors from the previous processing steps: the localization error due to the data alignment between ground truth and RFEC data, the inherent errors related to the technologies’ accuracy (laser scanner and RFEC tool), and the human accuracy for manually labeling the data.

While we propose an automatic approach to the data analysis, there is still a need for a pre-process of labeling for the training stage of the classifiers and the optimization of the parameters. Considering the size of the feature vector for the defect detection, a balanced dataset of one hundred points should be enough to train the classifier. For the segmentation algorithm, manual tuning of the parameters could be performed easily on a few samples. The physical meaning of these parameters makes this task trivial and therefore easy to use. Another limitation of the proposed approach is that it is designed only for RFEC tools equipped with several receivers distributed along the circumference. In fact, as we are using segmentation methods based on image processing algorithms, our approach would not work on the original sensor design proposed by MacLean [1], where only two coils (emitter and receiver) are used.

The proposed framework is applicable for automating the RFEC data analysis using a reliable and systematic approach. The availability of the defect shapes can certainly help in the decision-making of replacing used pipes. A direct example is to use the size and shape of a defect as an input to a stress analysis in order to study the probability of a pipe failure [41].

## Figures and Tables

**Table 1:** Confusion matrices are used to summarize the performance of a classification.

		Predicted Condition	
		***true***	***false***
**true**	***true***	tp	fn
**condition**	***false***	fp	tn

**Table 2:** Classifiers’ performances for the artificial dataset (ranked according to κ).

coef	*Precision*	*Recall*	*Accuracy*	κ
**SVM**	**1.00**	**1.00**	**1.00**	**1.00**
**Random Forest**	**1.00**	**1.00**	**1.00**	**1.00**
**Naive Bayes**	0.69	0.78	0.96	0.71
**Logistic Regression**	0.72	0.67	0.95	0.67

**Table 3:** Classifiers’ performances for the real dataset (ranked according to κ). The threshold for SVM has been chosen in order to have a precision equal to 1. Hence, the other metrics are slightly biased.

coef	*Precision*	*Recall*	*Accuracy*	κ
**SVM**	**1.00**	0.21	**0.97**	0.34
**Random Forest**	0.70	**0.42**	**0.97**	**0.51**
**Naive Bayes**	0.06	0.86	0.49	0.04
**Logistic Regression**	0.21	0.01	0.93	0.01

**Table 4:** Classifiers’ performances for defect detection in real dataset (ranked according to κ).

coef	*Precision*	*Recall*	*Accuracy*	κ
**SVM**	**0.96**	0.83	0.90	**0.84**
**Random Forest**	0.87	0.87	**0.92**	0.80
**Logistic Regression**	0.84	**0.89**	0.90	0.79
**Naive Bayes**	0.74	0.87	0.84	0.69

**Table 5:** Confusion Matrix for the SVM classification.

		Predicted Condition	
		**defects**	**outliers**
true	**defects**	298	59
condition	**outliers**	12	693

**Table 6:** Comparison of the segmentation performance on the artificial dataset (ranked according to F-score).

coef	*Precision*	*Recall*	*Accuracy*	*F*-Score
**Region Growing**	0.60	0.27	0.88	0.19
**Active Contour**	**0.77**	**0.69**	**0.99**	**0.66**

**Table 7:** Comparison of the segmentation performance on the real dataset (ranked according to F-score).

coef	*Precision*	*Recall*	*Accuracy*	*F*-Score
**Region Growing**	**0.63**	0.14	0.92	0.22
**Active Contour**	0.53	**0.48**	**0.93**	**0.48**
